# The lived experience of Sjögren’s Syndrome

**DOI:** 10.1186/s12903-016-0165-4

**Published:** 2016-02-01

**Authors:** Di Ying J. Ngo, William M. Thomson, Anita Nolan, Shelagh Ferguson

**Affiliations:** Department of Dental Surgery, Khoo Teck Puat Hospital, Singapore, Singapore; Department of Oral Sciences, Sir John Walsh Research Institute, Faculty of Dentistry, University of Otago, Dunedin, New Zealand; Department of Oral Health, Faculty of Health Sciences, Auckland University of Technology, Auckland, New Zealand; Department of Marketing, School of Business, University of Otago, Dunedin, New Zealand

**Keywords:** Dry mouth, Sjögren’s Syndrome, Lived experience, Patient perspectives

## Abstract

**Background:**

Sjögren’s Syndrome is an autoimmune exocrinopathy characterised by lymphocytic infiltration of exocrine glands in multiple sites, with dry mouth as a primary presenting symptom. Although quantitative studies have shown the negative impact of both dry mouth and Sjögren’s Syndrome on patients’ quality of life, no qualitative diary and interview study has been undertaken to examine the lived experience of dry mouth for Sjögren’s Syndrome sufferers. The aim of this qualitative study was to provide clinicians with insight into how dry mouth can impact on the daily lives of Sjögren’s Syndrome patients.

**Methods:**

The American-European Consensus Group (AECG) Revised International Classification criteria were used to identify participants from patients seen in an oral medicine clinic. After pilot study work to test the approach, the 10 main study participants were recruited. Diary entries and semi-structured interviews were used to explore how dry mouth affects their lives. Owing to the exploratory nature of the research, thematic content analysis was applied, allowing the themes to arise naturalistically from the data without bias or elicitation.

**Results:**

The data showed that it is unrealistic to understand the experience of a single symptom, but that the disease as a whole needs to be taken into perspective. The empirical evidence supported four main themes that depicted the lived experience of Sjögren’s Syndrome. These included: (1) the journey to diagnosis; (2) disease impact spectrum (of dry mouth amid other symptoms); (3) interactions with healthcare professionals; and (4) the positive coping process.

**Conclusions:**

The findings revealed patients’ perspectives on diagnosis, coping with dry mouth and Sjögren’s Syndrome, and interaction with healthcare professionals. Dry mouth is not a trivial symptom for Sjögren’s Syndrome sufferers; it has considerable impact on their day-to-day lives. Healthcare professionals need to understand patients as individuals in their environment in order to be part of the Sjögren’s journey.

## Background

Sjögren’s Syndrome has been defined as a chronic autoimmune disorder characterised by lymphocytic infiltration of exocrine glands in multiple sites [1]. In primary Sjögren’s Syndrome, dry mouth occurs with dry eyes only. In secondary Sjögren’s Syndrome, dry mouth and dry eyes occur in association with other autoimmune disorders such as rheumatoid arthritis or systemic lupus erythematosus [2]. The AECG Revised International Classification criteria for Sjögren’s Syndrome [3] are the diagnostic criteria most accepted in the clinical and research fields. Sjögren’s Syndrome has been found to be most common in middle-aged females, with a reported prevalence of 0.5 % to 1 % in the general population [4]. The aetiology of Sjögren’s Syndrome is multifactorial, involving a complex interplay of genetic, environmental, hormonal, and immunological factors [5]. The clinical manifestations of Sjögren’s Syndrome include dry mouth, dry eyes, and multiple extraglandular manfestations [6]. The most serious aspect of Sjögren’s Syndrome is the considerably higher risk of developing malignant non-Hodgkin’s lymphoma [7]. Dry mouth can manifest as xerostomia (a subjective sensation) or salivary gland hypofunction (an objective sign) [8, 9]. The clinical manifestations of dry mouth common to Sjögren’s Syndrome patients include multiple signs (such as dental caries, dental erosion, salivary gland swelling, mucositis, oral candida and ulcers) and symptoms (such as difficulty in swallowing, sensitivity to spicy foods, altered taste sensation, salivary gland pain, and speech difficulties) [10]. There is no cure for Sjögren’s Syndrome (or dry mouth), and the management involves dealing with the underlying systemic condition(s), alleviating symptoms, and instituting measures to prevent secondary complications [11–13].

**Fig. 1:**
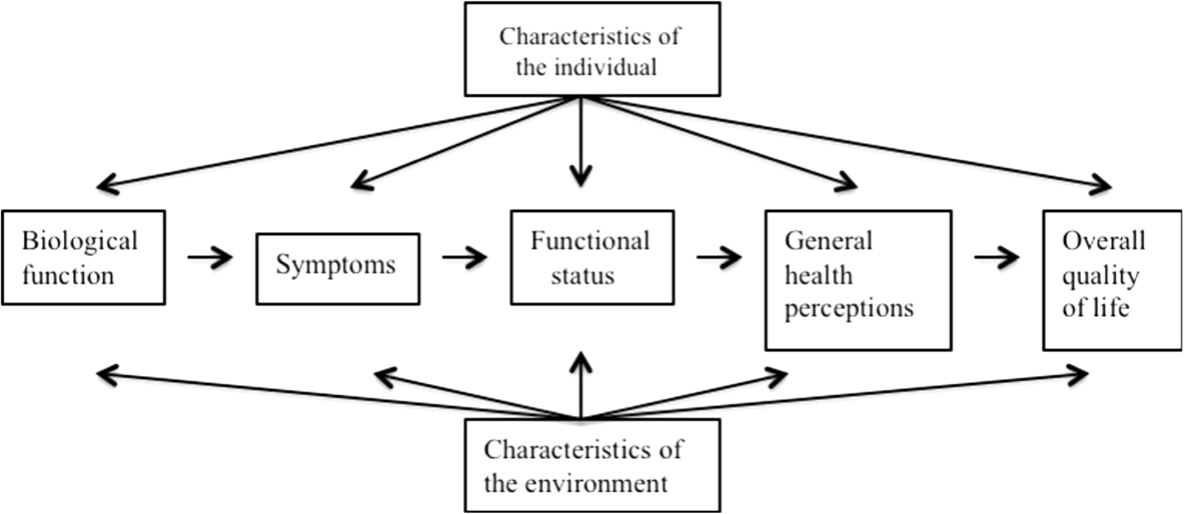
The Ferrans et al model [25]

The impact of dry mouth and Sjögren’s Syndrome goes beyond physical signs and symptoms. In accordance with Engel’s biopsychosocial model [14], the notions of health-related quality of life (HRQoL) and oral-health-related quality of life (OHRQoL) consider the physical, psychological, and social aspects of general and health. The notion of HRQoL considers that health perceptions integrate different components of health such as global perceptions of function and well-being. OHRQoL is a multidimensional construct that refers to the extent to which oral disorders disrupt an individual’s normal functioning. It can be considered to be a facet of HRQoL [15–18]. The 36-Item Short-Form Health Survey (SF-36) [19] is the most widely used multidimensional HRQoL measure [20]. The Oral Health Impact Profile [21] and the Geriatric/General Oral Health Assessment Index [22] are similar measures used to assess OHRQoL. Using these measures, dry mouth has been shown to negatively impact on quality of life [18, 23, 24]. Similarly, Sjögren’s Syndrome has been shown to negatively impact on the quality of life of its sufferers [25, 26]. Oral distress has been found to be prevalent and significantly greater in patients with Sjögren’s Syndrome, with marked effects on their HRQoL [27]. Wilson and Cleary proposed a conceptual model of patient outcomes to link clinical variables with HRQoL (29). The model encompasses disease, health, and quality of life outcomes depicting the causal relationships among the clinical levels: (1) physiological/biological variables; (2) symptom status; (3) functional status; (4) health perceptions; and (5) quality of life. The biopsychosocial model [14] is represented by the characteristics of the individual and environmental, as mediators of health outcome. The Ferrans et al. model (2005) [28] (Fig. 1) further simplified the model and provided theoretical background for each of its major components, hence, improving the understanding of the multidimensional components and pathways of HRQoL and OHRQoL [28, 29]. There are limitations as to how much such conceptual models can provide insight into patient perceptions in terms of: (1) difficulty in quantifying experience; (2) conceptualising disease experience affected by individual and environmental characteristics; (3) conceptualising changes in patient experience over time; and (4) conceptualising the effect of treatment. This suggests a need for a different research paradigm.

Qualitative studies on the impact of dry mouth and Sjögren’s Syndrome (separately) on individuals are scarce. Dry mouth has been found to cause debilitating impact on multiple domains of well-being (such as function and psychosocial), and there is a need to improve the understanding of health care professionals providing treatment for people afflicted by it [30–32]. Sjögren’s Syndrome has been shown to cause extreme fatigue that was debilitating and stressful, resulting in patients having to scale down their lives [33, 34]. Moreover, Sjögren’s Syndrome sufferers have been found to: live in hope of a future cure; be reliant on help from others; be hurting from physical and emotional suffering; and have their social lives hindered by the condition [35]. So far, no qualitative research has purported to study the impact of the dry mouth amid the range of systemic manifestations of Sjögren’s Syndrome. No HRQoL conceptual model has been applied to reveal the complex relationships behind the adverse impact of Sjögren’s Syndrome on quality of life. No research has yet provided a qualitative perspective to the Ferrans et al (2005) model [28]. There are gaps in understanding that can be approached only using qualitative methods. The intent of the qualitative inquiry was to understand the lived experience of dry mouth for Sjögren’s Syndrome patients. However, the findings reflect the participants’ reality that a sign or symptom cannot be singled out from a disease, and that the Sjögren’s experience had to be understood in a wholistic manner.

## Methods

Ethical approval for the study was obtained from the University of Otago Human Ethics Committee. The participant sample comprised volunteers from a pool of patients who had been seen by an oral medicine specialist (AN) in the University of Otago. The inclusion criterion was a positive diagnosis of Sjögren’s Syndrome (primary or secondary) according to the revised international classification criteria (AECG) [36]. In this criterion, there are both subjective and objective components to dry mouth. A participant may have either oral signs and/or symptoms. The exclusion criterion was the inability to commit to the data collection (that consisted of a 1-month diary entry and an interview). Contact was made by the primary researcher (DYJN) to explain the research process (using information sheets) and to obtain written and signed informed consent. This consent included permission to publish the research results. Pseudonyms have been used and each participant understands that every attempt will be made to preserve their anonymity.

A pilot study with 2 participants was carried out to test the diary (written/email) and interview processes. The written and email diary was a free choice option to tailor the research design to match the participants’ preferred mode of diarying. The diary data were collected either way. Two different diary methods were tested: (1) a guided, with-parameters approach; and (2) an unguided, minimal-instruction approach. An example of the guidance was on length of diary being one entry per day for a month. Some of the parameters included the condition’s effect on: (1) sleep patterns; (2) social events (such as shared meals); and (3) relationships with others. The pilot study identified that the first approach (1) for 1 month was appropriate for the participants to record reflections on and experiences pertinent to their disease experience [37]. During that month, rapport was maintained between DYJN and the participants using weekly (phone/email/direct) contact to discuss the diary entries. A list of core questions for each interview was derived from the preceding diary entry. The data from the pilot study were found to be rich, and so they were included in the complete study data collection.

Semi-structured interviews encouraged conversational interaction, with open-ended and non-leading questions that allowed participants to freely relate their thoughts on the effects of dry mouth and Sjögren’s Syndrome on their daily lives [38, 39]. The interviews (25-60 mins long) were audio-taped and conducted in a non-clinical environment, usually a meeting room in the Dental School. The types of questions asked are illustrated in Table 1 [40]. The list of questions evolved with each interview and narrowed down into the four main themes as data collection proceeded.

**Table 1:** Interview question types with examples (after Patton 2002)

Question type	Example
Behaviour or experience	Are there some foods you have to avoid?
Opinion or belief	Would you say that’s (dry mouth at night) the worst aspect of it impacting upon your life?
Feelings	How did you feel when they first told you that you had Sjögren’s Syndrome?
Knowledge	Did you know what it (Sjögren’s Syndrome) was?
Sensory	You mentioned food is sticking to your mouth… do you adjust to that?
Background	Do you think being a nurse affected how you got to your diagnosis…

The required sample size depended on the point of data saturation, whereby there was redundancy of themes (no new ones discovered) [41]. Data saturation was observed with the 9^th^ participant, and a 10^th^ participant was recruited as a check to confirm that the point of theme redundancy had been reached. All of the participants were middle-aged or older (corresponding to the age group in which Sjögren’s Syndrome is commonly diagnosed), with an age range from 46 to 86, and a mean age of 64. All were community-dwelling; 6 held regular jobs, and 4 were retired people. The female:male ratio was 9:1, reflecting the usual pattern seen in in Sjögren’s Syndrome prevalence [1].

The data transcription (diaries and interviews) was extensive (requiring 160 h in total). Each audio file was played back and manually transcribed by DYJN. Transcribed data included punctuation, expressions, laughter, and consistent speaker identification in order to minimise discrepancies in data transfer. The verbatim transcript was then checked by the co-authors, who then proceeded to analyse the data.

Data analysis commenced after each transcription. Thematic content analysis was applied [42]. The process involved the following steps: (1) familiarisation with the data: (transcription, reading, and noting initial ideas); (2) generating initial codes by systematic coding of data where the primary researcher and two supervisors reviewed the initial codes from the transcribed data (inter-researcher peer coding determined the reliability); (3) searching for themes, and collating data into potential themes (multiple mind-maps and discussions were employed in order to identify the different themes); (4) reviewing themes by checking them in relation to coded extracts; (5) defining and naming themes, thus generating clear definitions of each theme; and (6) reporting on findings, by relating the analysis to the research question and literature, thus producing a scholarly report of the analysis. Key emergent themes (based upon the existing literature) were identified through abstracting from specific analysis of the data and constant comparison between participants. These were then used to modify the framework for the interview questions used in the data collection process. The themes were considered in relation to the Ferans et al model [28, 29], with emphasis on the levels ‘characteristics of the individual’ and ‘characteristics of the environment’.

After each interview, the participant was asked to complete the Shortened Xerostomia Inventory (SXI) [43] in order to complement the diary and interview data. The scores were then computed, with a higher score indicating more severe dry mouth.

## Results

This section includes participants’ demographic data and SXI data. The rest is organised according to the four main themes derived from the qualitative analysis: (1) the journey to diagnosis; (2) interactions with healthcare professionals; (3) the disease impact spectrum; and (4) the positive coping process.

Table 2 summarises the participants’ demographic data, dry mouth status including the SXI data, and the length of time before Sjögren’s Syndrome diagnosis for each participant. The SXI scores ranged from 17 to 24, with a mean score of 20.9.

**Table 2:** Overview of participants’ demographic and disease characteristics

Demographic data			Dry mouth status (SXI score)	Number of years before Sjögren’s Syndrome diagnosis
Pseudonym	Age range	Gender		
Sandra	60s	Female	21	6
Amanda	50s	Female	24	6
Angela	60s	Female	19	28
Mary Anne	80s	Female	23	3
Joshua	50s	Male	18	4
Emily	60s	Female	21	6
Katherine	80s	Female	17	1
Maureen	60s	Female	24	4
Michelle	60s	Female	18	2
Rachel	40s	Female	24	7

### The journey to diagnosis

**Fig. 2:**
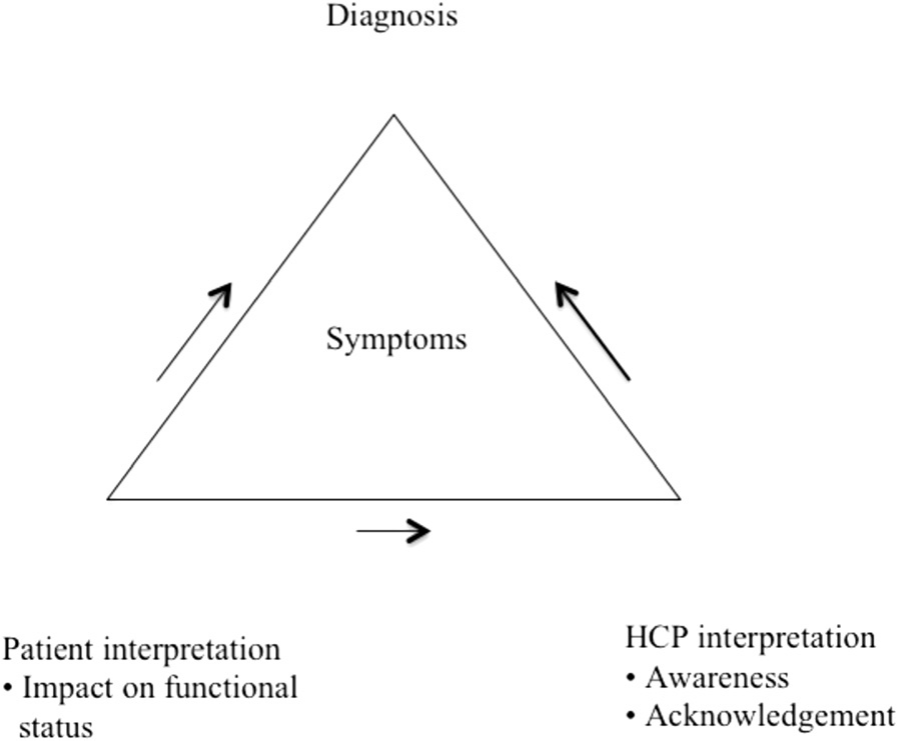
The journey to diagnosis

This theme marks the beginning of the Sjögren’s Syndrome (and associated dry mouth) experience. The pattern observed in the data included: (1) symptom interpretation by the patient; (2) symptom interpretation by the healthcare professional; and (3) heterogeneity in the medical routes that led to diagnosis (Fig. 2). The triangle symbolises the relationship among symptoms, patient, and healthcare professionals in the journey to diagnosis. In the centre of the triangle are the different symptoms that indicate an abnormal state.

From the participants’ perspectives, symptom interpretation was intertwined with impact on functional status. The initial physical symptoms among the sample of informants varied considerably, with a different combination for each participant, ranging from none (with an incidental finding of salivary gland hypofunction observed by a dental hygienist), intolerance to different foods, dry mouth, malar rash, bowel problems, swollen glands, pneumonia and sore joints, to general fatigue.

Michelle is a woman in her sixties who is fit and strong. She works on the farm and is responsible for physically demanding chores such as irrigation.

Michelle: *“During last winter I had trouble getting out of bed and getting clothes on and had no strength in my hands in the mornings, so I went back to the general practitioner as I was getting really concerned about being able to cope with irrigating as it is quite physical and I was in pain getting on the bike let alone doing anything.”*

Michelle interpreted her joint pain as significant because of its possibly adverse effect on her ability to work (and, hence, her financial stability). Other factors that had an effect on symptom interpretation included healthcare and disease experience. For example, those who had close family members who had suffered from serious illness (such as brain aneurysm or cancer) were naturally more worried that their symptoms were ominous.

How healthcare professionals interpreted participants’ symptoms affected the route to diagnosis. The healthcare professionals involved were a combination of the dental and medical team, including dental hygienists, dentists, oral medicine specialists, general practitioners, haematologists, opthalmologists, and rheumatologists. Their awareness and acknowledgement of Sjögren’s Syndrome symptoms have been shown to play a significant role. Having taken 5-6 years in her journey to diagnosis, Emily questioned general practitioners’ level of awareness of the initial Sjögren’s Syndrome symptoms.

Emily: *“Well, the thing is do the* general practitioners *know about it (Sjögren’s Syndrome)? Do they just think that somebody just gets flu and you know pneumonia or do they sort of think that it might be something, do they know what to look for because I was so long.”*

Emily’s general practitioner may not have been aware of the vague initial symptoms that were a manifestation of Sjögren’s Syndrome. Other than the level of awareness, symptom interpretation by healthcare professionals can only begin if they acknowledge those symptoms. Mary Anne, in her eighties, recalled that her early symptom of dry mouth was belittled and ignored, resulting in a *“battle”* to Sjögren’s Syndrome diagnosis. Her general practitioner sent her home with *‘you must be snoring at night’* and it took *“about 3 years to to finally get him to acknowledge that it was something”.* Dry mouth is an important symptom of Sjögren’s Syndrome that can often be ignored because of a lack of awareness of its significance.

The diagnosis was important because most of the research participants experienced a sense of relief to finally “*have a name for the disease*”. The range of times taken to diagnosis (1-28 years) by the current participants reflects the heterogeneous routes taken.

### Interactions with Healthcare Professionals

**Fig. 3:**
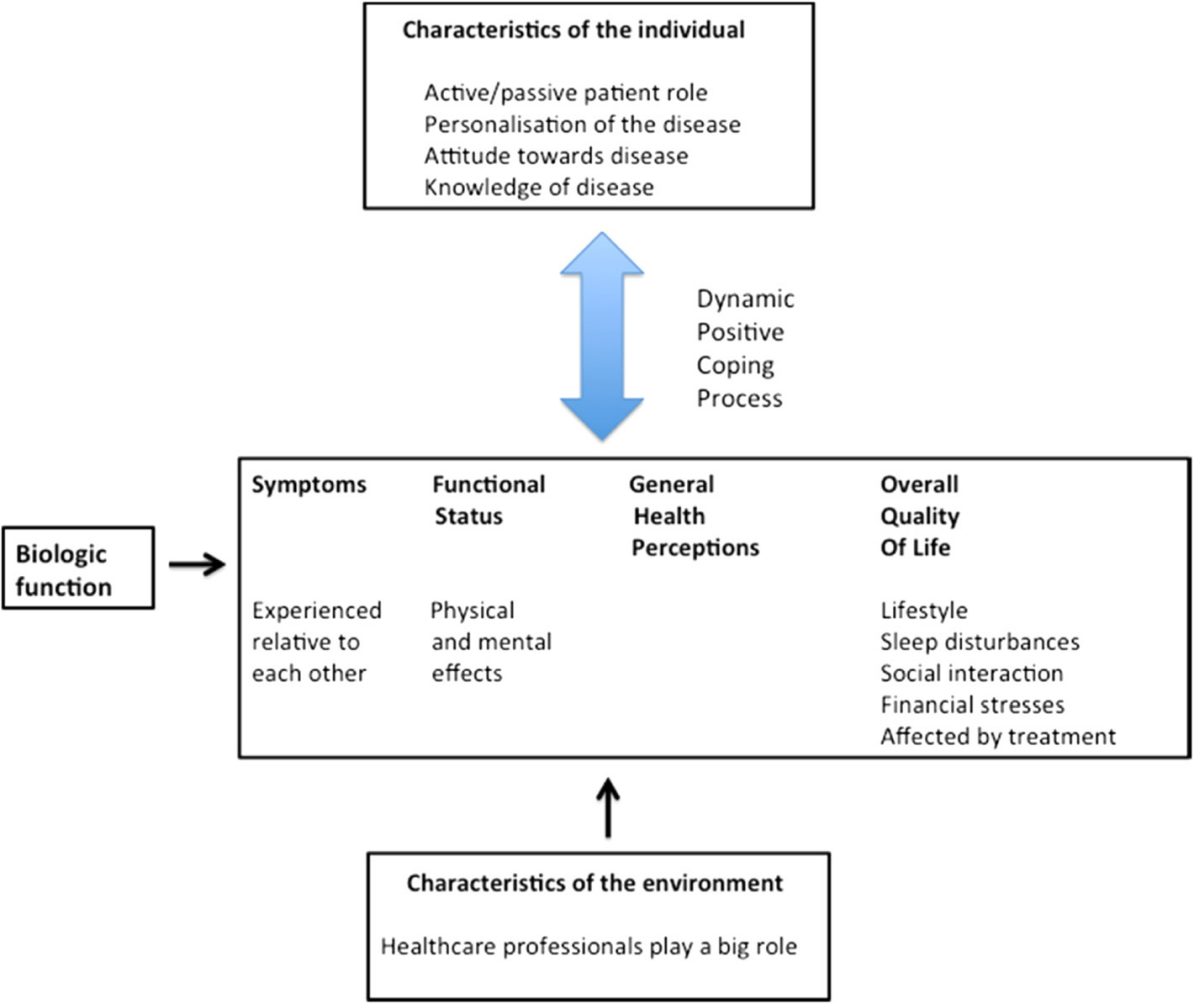
The participants’ view of the model of Ferrans et al [25]

This theme embodies the bi-directional link between the two levels ‘characteristics of the individual’ and ‘characteristics of the environment’ in the Ferrans et al model [28]. The patient role (active/passive) and the accessibility and empathy of healthcare professionals had important influences on this interaction (Fig. 3).

For the current research participants, there was a general dichotomy into passive or active patient roles. Those who played a more passive role tended to be less controlling, and more accepting of the prescribed treatment. By contrast, those in more active patient roles tended to chase after their own referrals, self-alter their prescription drugs, and participate actively in the medical decision-making. Rachel (who played an active patient role) experienced longstanding joint pain and opposed the idea of long-term medication.

Rachel: “*You’re chucking more pills down your throat.. I don’t wanna carry on doing that because every time I’ve gone to the* general practitioner *they just said, ‘oh well you know you’ve got your four pain pathways we can block every one of those.’ So you know, your anti-inflammatory, your paracetamol, your codeine, and so then you end up with more tablets.*”

Subsequently, Rachel (like most of the participants) began to actively explore complementary and alternative medicine, and started to see a nutritionist.

Moreover, healthcare professional accessibility and empathy improved the interaction between patients and healthcare professionals. The positive impact of accessible healthcare professional contact was evident in Michelle’s experience, where she expressed gratitude for being able to access a healthcare professional easily when she developed lumps in her neck (that were possible signs for Sjögren’s Syndrome-associated lymphoma).

Even though there was no cure for Sjögren’s Syndrome, the participants valued healthcare professionals as a source of empathy, because the sense of “*being understood*” through listening was important to them.

Mary Anne: “*It is so important that you are able to talk to them (healthcare professionals), I know at the moment there’s nothing that you can do for me! It doesn’t matter, but you were listening, you were not just tossing it*.”

The common pattern in the data emphasised the value of healthcare professional empathy through listening. The importance of the role of healthcare professionals is indicated in the ‘characteristics of the environment’ box in Fig. 3.

### Disease impact spectrum

This theme focuses on the participants’ view of the impact of Sjögren’s Syndrome (and dry mouth) on their quality of life, with reference to the Ferran’s et al model [28] (Fig. 3). The biological function was not important (hence represented in a small separate box), and the other distinct levels of the model were reconceptualised and merged into one level of disease impact. The findings described aspects of the participants’ perceptions, such as: (1) how the multiple Sjögren’s Syndrome symptoms related to each other; (2) how they experienced the less-tangible impacts of dry mouth; and (3) how treatment had negatively affected them.

Michelle: “*As soon as I get pain-free, I feel like I am a new person, I still got a dry mouth and a cough but I still feel like a new person*.”

Disease impact is complex, and each participant weighed and compared the different symptoms of Sjögren’s Syndrome. In quality of life measures (such as the SF-36), the two domains (physical and psychological) of functional status have commonly been scored separately. However, for Amanda, the two were intertwined: “*it was not just physical but it was mental tiredness as well*”.

The less-tangible impacts of dry mouth on quality of life were described in-depth by the participants. For Amanda, pain was inherent to eating (and brushing) because of the dryness associated with the skin around her mouth and her lips.

Amanda: “*It brings a lot of pain at times when you try and eat, clean your teeth and deal with the pain around my mouth when my skins flare up and my lips get so dry, they are constantly cracking. It is a constant battle trying to get everything lubricated. My water bottle is my best friend.*”

The other effects of dry mouth on the participants’ quality of life included: (1) food choices (because of reduced tolerance spiciness, rough texture, and acidity); (2) sleep disturbance; (3) social interaction and communication; and (4) financial stress. Emily was “*paying out thousands every year to a dentist*”. Finally, her private dentist suggested, “*Restorative dentistry at Dunedin Dental School*.” Emily’s response was: “*I haven’t got 30-40 thousand for this (restorative dental bills). Now what happens? Am I a casualty of hospital cuts? Perhaps removal of all teeth*?”.

The effects of treatment on the participants’ quality of life were because of the side-effects of medications (such as hydroxychloroquine, tricyclic antidepressants, and pilocarpine), and, interestingly, the side-effects of diagnostic procedures. For example, minor labial gland biopsy is one of the diagnostic procedures required by the AECG criteria for Sjögren’s Syndrome [3]. Post-biopsy numbness was a complication that adversely affected Angela’s functional status (through associated dribbling) and self-esteem. Angela was “*never an over-confident person”* and this complication “*dented it (her confidence) a wee bit more”*. The effect of Sjögren’s Syndrome on quality of life is not through the disease alone; the effect of treatment (and diagnosis) was significant to the participants, both physically and emotionally.

### The positive coping process

The process of coping with Sjögren’s Syndrome was a significant part of the disease experience for the participants, as illustrated in the blue arrow linking ‘characteristics of the individual’ and the ‘Symptoms, functional status, general health perceptions, and overal quality of life’ box in Fig. 3. The bi-directional arrow represents an ongoing process that is dynamic because of the changes that occured with time, and the cyclical nature of Sjögren’s Syndrome. The three areas (part of the individual’s characteristics) observed (not in any particular sequence) to be significant in the participants’ coping process were personalisation of, attitude towards, and knowledge of the disease.

The dynamicity of coping results from a change in illness perception, from initial uncertainty to progressive acceptance. Initially upon diagnosis, Michelle thought: “*Oh God, I’m going to have to live with a dry mouth for the rest of my life*.” With hindsight, she was able to articulate an altered illness representation: “*I’ve forgotten what it’s like not to have it (Sjögren’s Syndrome) now*.” The cyclical disease experience helped the participants to cope because they knew what to expect. Rachel expressed how she knew that, if she were “*having a flare-up*”, she could also “*have a remission (Rachel laughs) so it won’t be so bad (Rachel laughs)*.”

The personalisation coping process was evident from how each participant adopted a trial-and-error approach to discover which remedy worked best for him/her. Their preference for natural products and supplements was evident. Some particpants were creative; Mary Anne for example, found homemade blackcurrant juice to be effective for the dry mouth that was affecting her sleep at night. Moreover, the importance of personalising remedies was shown from how the same product (such as dry mouth gel) could produce opposite reactions in different participants.

For Michelle, a positive attitude was important because “*if you start thinking down that’s where you will head quickly*.” Another way of positive thinking was to take a step back and view Sjögren’s Syndrome relative to other worse conditions that were experienced by other members of the family or the public. Emily expressed the view that “*it (Sjögren’s Syndrome) is something you live with, there are people who are a hundred times worse than I am*”. A few of the participants expressed their delight at knowing that the tennis star Venus Williams also had Sjögren’s Syndrome. This allowed others to identify with their disease, and provided a language to discuss Sjögren’s Syndrome with friends (for a sense for support). Mary Anne felt that “*Venus helped terrifically to make it (Sjögren’s Syndrome) known*”. Increased public knowledge and awareness of Sjögren’s Syndrome therefore allowed it to be less of a “*hidden disability*”. Some participants found the research process itself to be an altruistic act with positive influences on their self-esteem.

In the current study, the acquiring of knowledge (about Sjögren’s Syndrome) helped the research participants in understanding their condition. Some of it was intimidating, and some was helpful. The Internet was a source of knowledge for 9 out of the 10 research participants (or their families). When the information was presented as ‘worst case scenarios’, it was found to be intimidating.

Angela: “*Some of the things on the computer they are pretty horrific, you know you get a lot of the worst case scenario things and so I really thought, well I don’t know that I actually really need to know all just at this point in time*..”

## Discussion

The discussion which follows is divided into a summary of the study’s findings, limitations and strengths, and contributions, along with some consideration of future directions for research in this field.

This study used qualitative diaries and interviews to reveal in depth the lived experience of Sjögren’s Syndrome among 10 participants. Their reports of the severity of their dry mouth experience were supported by a mean SXI score that was twice that observed in any of the older samples used in the SXI validation study [43]. Overall, the findings fell into the 4 main themes of the journey to diagnosis, interactions with healthcare professionals, the disease impact spectrum, and the positive coping process. These themes described the overall picture of the lived experience of SS, beginning from finding a name for the disease, to what was important while relating to healthcare professionals, to how different aspects of Sjögren’s Syndrome impacted on quality of life, and the ongoing strategies to deal with the chronic disease.

Turning to the study’s limitations, there were 9 potential participants who either did not consent or did not follow through with the data collection. This limitation was because of personal preferences (such as a disinterest in keeping diaries and undergoing interviews) or more important priorities (such as workload or a close family member with a recent cancer diagnosis). The nature of that “undiscovered” information and knowledge is unknown, but it may have been valuable. There is a possibility that those who coped more positively were more willing to talk about their disease, hence creating a bias in terms of knowledge.

In qualitative research, there is the potential for the researcher’s influence to bias the data. The methods used to overcome this limitation included: maintaining a purely researcher-participant relationship (instead of a clinician/researcher-participant relationship); using the Ferrans et al model [28] as a reference framework; and employing inter-researcher peer coding in order to enhance the trustworthiness of the data. All of these strategies seek to create an account of method and data that can stand independently and to produce a plausible and coherent explanation of the phenomenon under scrutiny [44].

There is a possibility that the research participants tended to provide data that they perceived to be required of them, as mentioned by Michelle: “*I wasn’t too sure I was giving you the right information*..”. This phenomenon has been described as the “Hawthorne effect”, referring to the tendency of participants to modify their behavior because of their awareness of being under study [45]. This effect is a social acquiescence bias as the informant would like to provide to the interviewer, a healthcare professional, the ‘right’ answer. This limitation was addressed by reassuring the participants that there were no ‘correct’ answers and that any aspect of their experience was relevant to the investigation.

Turning to the strengths of the study, the data collection was undertaken after an initial meeting or phone call, in order to establish rapport between the primary researcher and each participant. This rapport was intended to enhance the participants’ ability to share their experiences freely. Both the interviews and the transcription were performed by the primary researcher. This process allowed for the interpretation of any non-verbal communication and the familiarisation with the entire data set [42]. The trustworthiness of the study was ensured by the analysis being conducted by a group (a process also known as ‘peer coding’). Moreover, triangulation was undertaken by using two different data collection methods to answer the same research question; these were the diary entries and the interviews, with this dual approach intended to enhance the data trustworthiness [44].

The transferability of a qualitative research project is the generalisability of the findings to other situations and contexts. A recent qualitative study by Owens et al (2014) in Sheffield (UK) found dry mouth to impair speech, sleeping, working, eating and self-care [32]. The participants’ perspectives in the current research were in line with the experiences of their sample. A Swedish qualitative study by Folke (2009) identified xerostomia as an ‘aggravating misery’ and revealed the participants’ main concerns to be ‘professional consultation’, ‘search for affirmation’ and ‘social withdrawal’. There were some overlaps in the content of the themes between that Swedish study and the present study. It is therefore appropriate to assert a degree of transferability of the current research findings to other dry mouth sufferers.

Turning to this study’s contribution, the mean SXI score for the participants was more than twice that of any of the samples used in the international validation study for the SXI (using older people). This disparity illustrates the severity of dry mouth experienced by these Sjögren’s Syndrome patients because xerostomia is already known to be more severe (and of higher prevalence) in older people than among younger people [46]. The analysis of the SXI scores has provided a quantifiable description of the dry mouth experiences of these participants, complementing the descriptions made in the themes. The participants’ perceptions provided important insight into the patient experience of living with Sjögren’s Syndrome (and dry mouth).

The results showed that the dry mouth experience in Sjögren’s Syndrome cannot be understood on its own, but the disease needs to be understood in a wholistic manner. In the *journey to diagnosis* theme, the participants evaluated their symptoms by making judgments about the severity and effect on their lives [47]. They questioned the awareness and acknowledgement of the initial Sjögren’s Syndrome symptoms by healthcare professionals. The range of initial Sjögren’s Syndrome symptoms is broad and can be vague, causing healthcare professionals difficulty in identifying presenting symptoms as a manifestation of the condition [35]. General practitioners played a key role in directing the diagnosis of Sjögren’s Syndrome because they were the first port of call. It is therefore important to improve general practitioners’ awareness of Sjögren’s Syndrome manifestations (such as dry mouth). This improvement can be achieved through continuing professional development involving collaboration between dental and medical colleagues. Early diagnosis and appropriate management are essential for optimal health outcomes in Sjögren’s Syndrome [48]. The range of times taken to diagnosis (1-28 years) puts into context the findings from an American study investigating the health experience of 277 Sjögren’s Syndrome patients, where the mean time to diagnosis was reported to be 7 years. This delay was attributed to the non-specific nature of the presenting symptoms, or to poor physician awareness of Sjögren’s Syndrome [26].

The findings in the theme *interactions with healthcare professionals* are supported by the exisiting literature, where healthcare professionals are part of the social environment that can influence health [49]. Bodenheimer et al (2002) described two types of patient-healthcare professional relationships: the traditional relationship, and the patient-professional partnership. In the traditional relationship, the patient is more passive, and agrees to the healthcare professional’s treatment prescription. In the patient-professional partnership, the patient plays an active role in care collaboration and self-management education [50]. The participants tended toward two patient roles (the more “active” or “passive”) [51] and these affected their expectations of healthcare professionals. It would be useful for healthcare professionals to increase patient involvement, because active patient participation in medical decision-making has been shown to improve health outcomes [52]. Most of the participants (including those who played either active or passive roles) sought alternative medicine support at some stage. In chronic diseases, this response can be a way of attempting to alter the illness trajectory by finding someone who might provide an alternative picture of it [53]. While healthcare professionals prescribed medications in the faith that it would positively influence the participants’ quality of life, the latter may perceive the opposite. The patient-physician relationship can impact on quality of life, it is therefore important to understand the nature of the relationship [54]. Electronic consultations [55] may be a useful avenue to increase healthcare professional accessibility and improve effective patient-physician interaction in collaborative Sjögren’s Syndrome care. Healthcare professional empathy that is reflected through listening is important to patients because they perceive it to be an essential component of clinical data gathering and diagnosis, a healing and therapeutic agent, and a means of fostering and strengthening the doctor–patient relationship [56].

The *disease impact spectrum* theme reflected how the participants perceived the impact of dry mouth as a subset of the other consequences of Sjögren’s Syndrome. In the existing literature, disease impact of Sjögren’s Syndrome been considered in a segregated manner (divided, for example, into the domains of dry eyes, dry mouth, or employment status) [57–59]. However, during the description of their Sjögren’s Syndrome experiences, every participant described several symptoms together (such as dry eyes, fatigue, sore joints, and lowered immunity). There will be a comparison and prioritising of symptoms as patients self-manage multiple chronic conditions [60]. This patient perspective challenges the usual view adopted by specialists in various fields, who tend to focus on the ‘area of the body’ that has been referred to them. Considering the participants’ experience of the impact of dry mouth on food choices and nutrition, it would be prudent to provide dietitians advice for newly-diagnosed SS patients. An interprofessional collaborative team approach to treating and managing Sjögren’s Syndrome patients and their dry mouth would be beneficial because of the interwoven disease impact spectrum. Medical intervention (medications and biopsy) had negative effects on the quality of life of the participants. This is an important area for clinicians to note, and it has yet to be explored in the literature.

In the *positive coping process* theme, the dynamicity displayed in the participants’ coping response reflects the phenomenon of “response shift”, whereby individuals adapt and reappraise their situation over time [61]. This phenomenon may offer an explanation for the changes with time that can be observed when using quantitative HRQoL or OHRQoL measures during the trajectory of a chronic disease. It is important for healthcare professionals to appreciate the dynamics of the coping process in order to provide more support during the early stages. Understanding how patients personalise their Sjögren’s Syndrome coping style should facilitate patient education for self-management in order to enhance a personalised problem-solving approach [62]. The psychosocial aspect of the participants’ coping processes supports the existing finding that chronic diseases carry important psychological and social consequences that demand significant adjustment. Seeking social support is an active coping approach with positive outcomes [62]. Moreover, the participants’ device of comparing their current circumstances with a hypothetical worse situation is a way of using positive illusions to cope with adversity [63]. Venus Williams having Sjögren’s Syndrome may have helped in the coping process for the participants in alleviating the sense of loneliness and lack of support that has been noted in those suffering from a less-known disease [64]. The view of research participation as an altruistic act to boost self-esteem corresponds to findings from UK research involving qualitative interview findings, where potential therapeutic effects on emotions were observed in participants [65]. The positive attitudes adopted by the participants comprise a form of a cognitive strategy to counteract the negative effect of illness on their well-being [66]. The participants (and their families) mostly acquired knowledge from the Internet to improve their cognition of Sjögren’s Syndrome. The ‘worst case scenarios’ found on the Internet were found to be intimidating. Healthcare professionals should perhaps inform patients of suitable information sources such as reliable websites (including social media sites such as the Sjögren’s Syndrome foundation Facebook group), books, or societies. Identifying factors that improve the coping process will aid in achieving the collaborative care and self-management education that is part of the preferred patient-physician partnership in dealing with chronic diseases [50].

The theoretical contribution includes added insight into the bidirectional relationship between the components ‘characteristics of the individual’ and ‘characteristics of the environment’ in the Ferrans et al model (2005) [28]. Nonetheless, there were pertinent patterns in the participants’ Sjögren’s Syndrome experience that were not completely described by the model, and an original model (Fig. 2) was drawn from the empirical findings. The re-conceptualisation of the Ferrans et al model (2005) [28] from the patients’ perspective (Fig. 3) is a significant contribution because no other study has provided a qualitative perspective to this model.

More qualitative research in different samples, cultures, or geographic locations can further test the conceptual model [28] and provide more insight into HRQoL and OHRQoL, which are dynamic and complex concepts. The subjective component of dry mouth (xerostomia) means that the insights from this research are applicable in understanding trial-related changes in the dry mouth (and Sjögren’s Syndrome) symptom burden, or for the comparison of symptom burden between intervention arms in clinical trials, and may provide sufficient self-reported data for clinical trial consumers to make treatment choices or to evaluate new therapies [67]. Moreover, the influence of individual and environmental characteristics should be included in current HRQoL/OHRQoL measures. An example of an item could be “How would you rate dry mouth amidst your other symptoms?” or “Has your healthcare professional been helpful in co-managing your dry mouth with you?”. The pertinent points from the unique dry mouth experience of Sjögren’s Syndrome patients may be compiled into an original Sjögren’s Syndrome-specific dry mouth inventory. Given the severity of dry mouth (as represented by the SXI score), this group of patients definitely requires more understanding and assessment of their dry mouth status. Advocacy for funding for dry mouth sufferers has been validated by the research findings [68].

## Conclusions

The findings revealed patients’ perspectives on diagnosis, coping with dry mouth and Sjögren’s Syndrome, and interaction with healthcare professionals. Dry mouth is not a trivial symptom for Sjögren’s Syndrome sufferers; it has considerable impact on their day-to-day lives. Sjögren’s Syndrome patients must be examined in the context of their individual characteristics (such as personality, lifestyle, and perceptions) and the environment in which he/she lives (and which healthcare professionals are a part). Healthcare professionals need this understanding in order to be part of the Sjögren’s journey.

## Abbreviations

AECG: American-European Consensus Group
HRQoL: Health-related Quality of Life
OHRQoL: oral-health-related quality of life
SF-36: short-form health survey
SXI: Shortened Xerostomia Inventory
